# Tuberculosis of the Tibial Plateau Mimicking a Giant Cell Tumor: A Case Report

**DOI:** 10.7759/cureus.43785

**Published:** 2023-08-20

**Authors:** Achref Miry, Mohammed Tbouda, Youssef Y Bouhajeb, Sanae Abbaoui

**Affiliations:** 1 Pathology, Faculty of Medicine and Pharmacy of Agadir, Agadir, MAR; 2 Pathology, Souss Massa University Hospital, Agadir, MAR; 3 Pathology, Mohammed V Military Hospital, Agadir, MAR; 4 Pathology, Ibn Rochd Pathology Center, Agadir, MAR

**Keywords:** giant cell tumor, medial tibial plateau depth, tibia non-union, calcifying fibrous pseudotumor, genital tuberculosis, tuberculous osteomyelitis

## Abstract

Tuberculous osteomyelitis is infrequent and occurs most often in the femur, the tibia, and the small bonne of hands and feet. Herein, we report a 39-year-old female who presented with chronic pain and motion range reduction of the left knee joint for two years. A knee radiograph revealed a geographic lytic lesion of the epiphyseal and diaphyseal region of the tibia mimicking giant cell tumor (GCT). A minimally invasive biopsy of the lytic lesion was performed, and pathological assessment revealed granulomatous inflammation made of numerous caseating necrotizing epithelioid and giant cells granulomas, diagnostic of tibial plateau tuberculosis. This case underscores the importance of taking tuberculosis into consideration in lesions mimicking GCTs in the tibial plateau, especially in endemic regions.

## Introduction

Tuberculous osteoarthritis is an uncommon infection although it occurs in higher frequencies in endemic countries such as ours (Morocco), namely in the form of tuberculous osteitis, with an incidence of 19% among all tuberculosis cases, typically following Pott's disease and tuberculous arthritis [1,2].

This condition refers to a range of pathological symptoms that occur when mycobacterium tuberculosis affects the skeletal and joint structures of the musculoskeletal system. When both bone and articulation are affected, the disease is called tuberculous osteoarthritis. However, tuberculous osteitis does not involve the joints [1]. The spread of TB to joints and bones occurs through hematogenous dissemination. TB can manifest in various presentations in bone, and the radiographic characteristics are frequently not specific [1].

Herein, we report a 39-year-old woman who presented with chronic pain and motion range reduction of the left knee joint for two years. A knee radiograph revealed a geographic lytic lesion of the epiphyseal and diaphyseal region of the tibia mimicking giant cell tumor (GCT). A minimally invasive biopsy of the lytic lesion was performed, and pathological assessment revealed granulomatous inflammation made of numerous caseating necrotizing epithelioid and giant cells granulomas, diagnostic of tibial plateau tuberculosis.

## Case presentation

We report a 39-year-old woman, with unremarkable medical history, who presented with insidious chronic pain and motion range reduction of the left knee joint for two years, with no general deterioration. The patient was unable to walk or stand. She reports no traumatic history.

Physical examination revealed an afebrile patient, with the presence of a mild fluctuation of the left pretibial region with normal-looking skin and no signs of tuberculous involvement, namely no adenopathies were clinically identified. No synovial thickening or systemic symptoms was found. The patient’s blood count and erythrocyte sedimentation rate were normal and were, respectively, at 6 x 10^9^/L and 5 mm/hr, with discrete C-reactive protein (CRP) elevation at 1.4 mg/dL with a normal value being <0.3 mg/dL.

**Figure 1:**
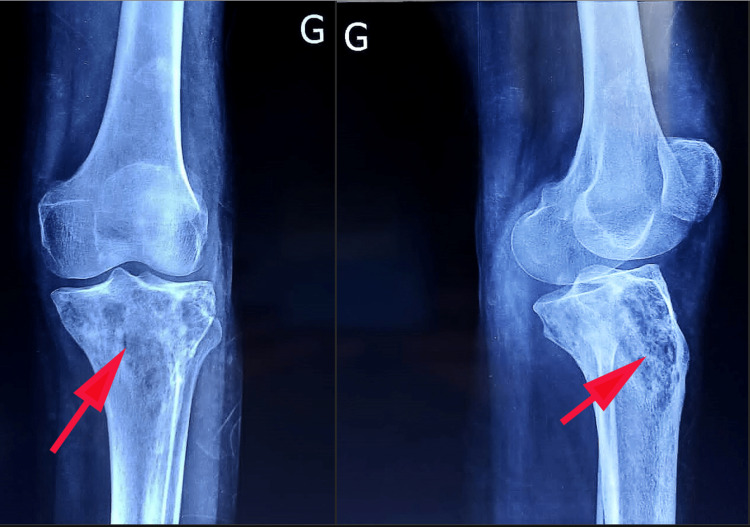
Left knee radiograph revealed a geographic lytic lesion of the epiphyseal (left) and diaphyseal (right) region of the left tibial plateau (red arrows).

A knee radiograph revealed a geographic lytic lesion of the epiphyseal and diaphyseal region of the left tibial plateau. No periosteal reaction was observed. This raised suspicion of osteosarcoma or a GCT considering the age of the patient and the proximity to the knee (Figure 1).

**Figure 2:**
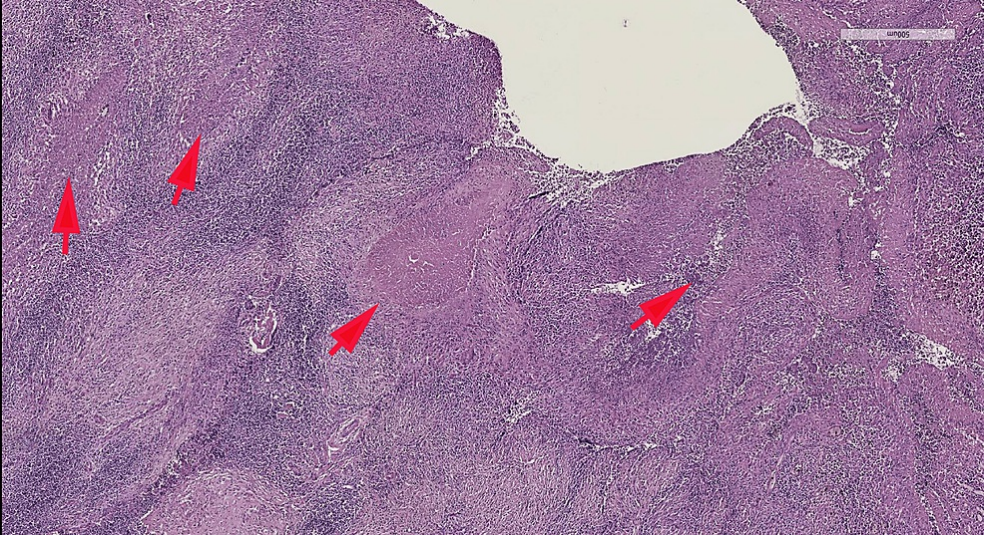
Microphotography showing the presence of granulomatous inflammation made of numerous caseating necrotizing epithelioid and giant cells granulomas (red arrows) (HE, 40x).

**Figure 3:**
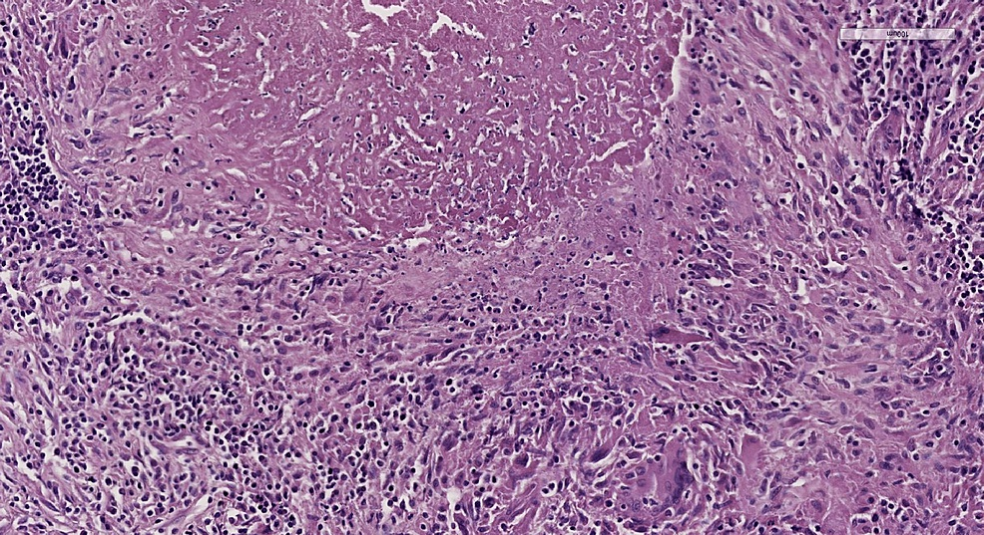
Microphotography at higher magnification revealing the presence of giant cells, epithelioid cells and the caseating nature of the central necrosis (HE, 200x).

The magnetic resonance imaging revealed a pseudo-tumoral appearance with the presence of a lesion suggestive of malignancy in the external part of the tibial plateau. The lesion was responsible for cortical rupture and contiguous extension toward surrounding soft tissue. A minimally invasive biopsy of the lytic lesion was performed, and pathological assessment revealed granulomatous inflammation made of numerous caseating necrotizing epithelioid and giant cells granulomas, diagnostic of tibial plateau tuberculosis (Figures 2, 3).

The patient was treated with anti-tuberculosis medication for a duration of nine months (Isoniazid 300 mg OD, Rifampicin 600 mg OD, Pyrazinamide 1500 mg OD, Ethambutol 1000 mg OD, and Pyridoxine 10 mg OD). During this period, she was not allowed to bear weight on the left side for six months. Follow-up after a period of four months revealed good clinical progress and bone reconstruction on the standard x-ray.

## Discussion

Osteoarticular tuberculosis and tuberculous osteitis are frequently reported in countries with underdeveloped and developing healthcare systems such as in our country (Morocco). Osteoarticular tuberculosis primarily affects the joint system and should be differentiated from tuberculous osteitis (as observed in our case), which does not involve the joints. Tuberculous osteitis, also called tuberculous osteomyelitis is a common occurrence in our country, typically appearing after Pott's disease and tuberculous arthritis. In a study by Martini [3], a total of 111 cases were reported over a period of 16 years, which reflects an incidence of 19% among all tuberculosis cases. Tuberculous osteomyelitis primarily affects young adults in Africa and the elderly or immunocompromised individuals in Western countries. There is a higher prevalence among females, accounting for 65% of the cases [3,4]. The spread of TB to joints and bones occurs through hematogenous dissemination. Predisposing factors consist of trauma, immunosuppressive factors like alcoholism, corticosteroid therapy, and HIV/AIDS [3,5].

The diagnosis of osteoarticular TB is often delayed due to the presence of nonspecific symptoms, which can resemble other conditions [6]. The symptoms are usually insidious. General symptoms are uncommon, except in cases of multifocal forms. The present case did not have any general symptoms. The high occurrence of fistulas (81%) in Martini's study can be attributed to delayed diagnosis [3]. Swellings and abscesses are less frequent compared to osteoarthritis [4,5].

Typical radiological characteristics of TB include juxta-articular osteopenia, narrowing of the joint space, and erosions [7]. Some of the most commonly described conditions that mimic TB are pigmented villonodular synovitis [8], pyogenic arthritis, tumors, and inflammatory disorders [9]. In 1920, Jungling described a distinct lacunar image surrounded by fine osteocondensation, termed “multiple pseudocystic tuberculous osteitis” [3]. The presence of bone sequestra forming a bell-shaped appearance can pose a differential diagnosis challenge with bone tumors [5]. The presence of lacunar images may indicate multiple myeloma or metastatic lesions, particularly in elderly individuals [5]. Other infections should also be considered in front of lacunar bone lesions, such as parasitic infections (histoplasmosis, and less commonly cryptococcosis and actinomycosis) [1]. Bone sarcoidosis, in its pseudocystic lacunar form, can be perplexing, even during the histopathological examination, and should also be considered among differential diagnoses [3,5]. In our case, the visualized lesion was highly suggestive of a neoplastic lesion, and osteosarcoma and an aggressive GCT were considered, especially since cortical effraction and soft tissue invasion were observed. Aggressive GCTs are associated with a broader zone of transition [9]. Osteoclastomas commonly occur in the knee (50%-65%), followed by the distal radius (10%-12%), sacrum, and vertebrae. They typically manifest in adulthood, typically between the ages of 20 and 50. Radiographically, they exhibit characteristic features such as close proximity to the closed growth plate, adjacency to the articular surface, a non-sclerotic margin, and an eccentric appearance [10].

The treatment primarily relies on medical management using anti-tuberculosis medications. The standard duration of treatment is one year, but more recently, shorter regimens have shown efficacy and benefits [1]. Immobilization is implemented to alleviate pain and prevent or correct deformities. Surgery plays a significant role not only in performing biopsies or draining large abscesses but also in preventing or correcting deformities [1].

## Conclusions

Osteoarticular tuberculosis and tuberculous osteitis are frequently reported in countries with underdeveloped and developing healthcare systems such as in our country (Morocco). Our case emphasizes considering the diagnosis of tuberculosis as a possible differential diagnosis for GCT and shows that similar cases should be explored through various diagnostic methods including radiological and bacteriological means, notably indicating the search of Koch's bacillus during direct examination and after culture, and histopathologically, notably suggesting a possible diagnosis of tuberculosis to the pathologist.
